# Deficiency of GD3 Synthase in Mice Resulting in the Attenuation of Bone Loss with Aging

**DOI:** 10.3390/ijms20112825

**Published:** 2019-06-10

**Authors:** Shoyoku Yo, Kazunori Hamamura, Yoshitaka Mishima, Kosuke Hamajima, Hironori Mori, Koichi Furukawa, Hisataka Kondo, Kenjiro Tanaka, Takuma Sato, Ken Miyazawa, Shigemi Goto, Akifumi Togari

**Affiliations:** 1 Department of Pharmacology, School of Dentistry, Aichi Gakuin University, 1-100 Kusumoto-cho, Chikusa-ku, Nagoya 464-8650, Japan; ag163d13@dpc.agu.ac.jp (S.Y.); lb_hidenori@yahoo.co.jp (Y.M.); Hamajima.k0329@gmail.com (K.H.); hironori128@gmail.com (H.M.); KondoH@dpc.agu.ac.jp (H.K.); kenjiro1@dpc.agu.ac.jp (K.T.); togariaf@dpc.aichi-gakuin.ac.jp (A.T.); 2 Department of Orthodontics, School of Dentistry, Aichi Gakuin University, Nagoya 464-8650, Japan; tk-mail@dpc.agu.ac.jp (T.S.); miyaken@dpc.aichi-gakuin.ac.jp (K.M.); shig@dpc.agu.ac.jp (S.G.); 3 Department of Biomedical Sciences, Chubu University College of Life and Health Sciences, Kasugai, Aichi 487-8501, Japan; koichi@isc.chubu.ac.jp

**Keywords:** glycosphingolipids, gangliosides, glycosylation, bone metabolism

## Abstract

Gangliosides are widely expressed in almost all tissues and cells and are also considered to be essential in the development and maintenance of various organs and tissues. However, little is known about their roles in bone metabolism. In this study, we investigated the effects of genetic deletion of ganglioside D3 (GD3) synthase, which is responsible for the generation of all b-series gangliosides, on bone metabolism. Although b-series gangliosides were not expressed in osteoblasts, these gangliosides were expressed in pre-osteoclasts. However, the expression of these gangliosides was decreased after induction of osteoclastogenesis by receptor activator of nuclear factor kappa-B ligand (RANKL). Three-dimensional micro-computed tomography (3D-μCT) analysis revealed that femoral cancellous bone mass in GD3 synthase-knockout (GD3S KO) mice was higher than that in wild type (WT) mice at the age of 40 weeks, although there were no differences in that between GD3S KO and WT mice at 15 weeks old. Whereas bone formation parameters (osteoblast numbers/bone surface and osteoblast surface/bone surface) in GD3S KO mice did not differ from WT mice, bone resorption parameters (osteoclast numbers/bone surface and osteoclast surface/bone surface) in GD3S KO mice became significantly lower than those in WT mice at 40 weeks of age. Collectively, this study demonstrates that deletion of GD3 synthase attenuates bone loss that emerges with aging.

## 1. Introduction

Gangliosides, which are sialic-acid-containing glycosphingolipids, are highly expressed in the nervous tissues of vertebrates [1] and have been considered as being involved in the regulation of nervous systems [2,3,4,5]. Recent studies of mutant mice lacking various gangliosides have revealed that gangliosides are essential in the maintenance of the integrity of nervous tissues and spermatogenesis, as well as the homeostasis of organisms [6,7,8]. GM3-only mice (double knockout mice lacking GM2/GD2 synthase and GD3 synthase genes) have exhibited severe neurodegeneration caused by excessive complement activation in the brain and spinal cord [9,10]. Mice lacking GM2/GD2 synthase gene have shown that gangliosides also play important roles in the transport of testosterone to the seminiferous tubules and bloodstream from Leydig cells [6].

In particular, b-series gangliosides, including GD3, GD2, GD1b, GT1b, and GQ1b, all of which contain α-2,8 sialic acid, have been considered as playing crucial roles in the maintenance of the sensory nervous system, neuroregeneration of hypoglossal nerves after injury, and secretion of leptin in adipose tissues [7,11,12]. On the other hand, these gangliosides have been identified as cancer-associated antigens, and are known to enhance tumor phenotypes, such as cell proliferation, and invasion in melanomas, osteosarcomas, and small cell lung cancers [13,14,15].

Some studies have reported that gangliosides are expressed in bone marrow mesenchymal stem cells (MSCs) and osteoblasts [16,17,18,19,20]. It has been documented that a-series ganglioside GD1a contributes to the regulation of osteoblast differentiation in MSCs [16,17,18]. However, all these studies were conducted only in cultured cell systems and there have been no reports on the involvement of gangliosides in bone metabolism in animals.

In this study we examine expression levels of b-series gangliosides (GD3, GD2, GD1b, and GT1b) in MC3T3 E1 mouse osteoblast-like cells, RAW264.7 pre-osteoclasts, and primary cultured pre-osteoclasts derived from mouse bone marrow cells using flow cytometry. Furthermore, in order to examine the involvement of b-series gangliosides in bone metabolism in vivo, we analyzed femoral cancellous bone mass in wild type and GD3 synthase-knockout mice using three-dimensional micro-computed tomography (3D-μCT). We also employed bone histomorphometric analyses using tartrate-resistant acid phosphatase (TRAP) and hematoxylin and eosin (HE) staining.

We report here that deletion of GD3 synthase in mice results in the suppression of bone resorption. To our knowledge, this report suggests, for the first time, that gangliosides are involved in bone metabolism in animals.

## 2. Results

### 2.1. No Expression of b-Series Gangliosides and GD3 Synthase Gene in Osteoblasts

**Figure 1:**
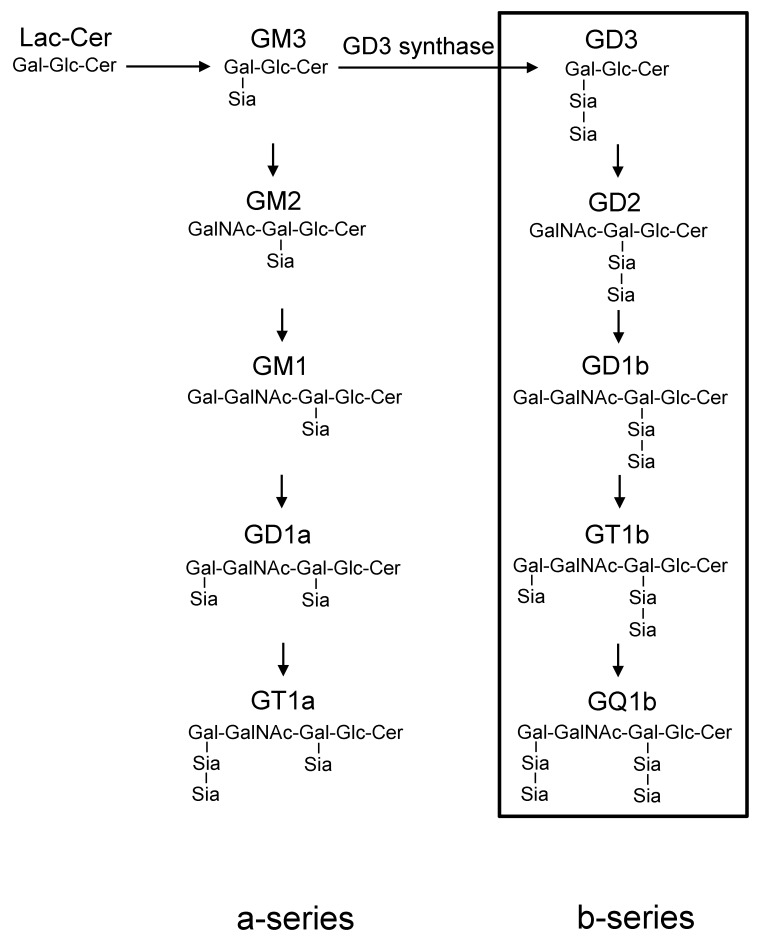
Synthetic pathway of gangliosides and the effects of targeted disruption of GD3 synthase. All b-series gangliosides (GD3, GD2, GD1b, GT1b, and GQ1b) in the box were deleted in GD3 synthase-knockout (GD3S KO) mice.

**Figure 2:**
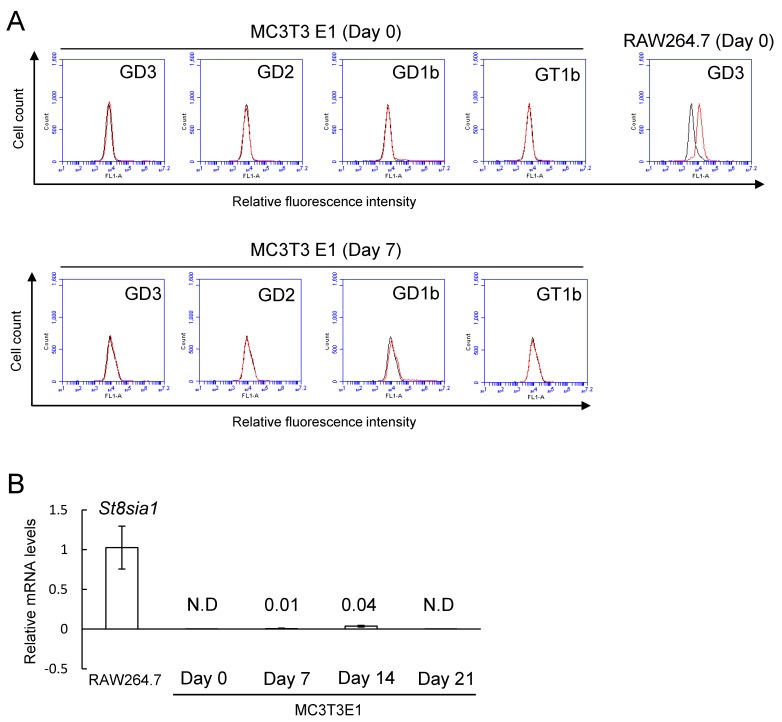
No detection of b-series gangliosides (GD3, GD2, GD1b, and GT1b) and GD3 synthase gene (*St8sia1*) in MC3T3 E1 cells. (**A**) Expression of b-series gangliosides in MC3T3 E1 cells on days 0 and 7 after induction to mature osteoblasts by flow cytometric analysis. Expression of GD3 in RAW264.7 cells without induction of osteoclastogenesis (Day 0). Red line: anti-gangliosides (GD3, GD2, GD1b, or GT1b) monoclonal antibodies (mAbs) (+), gray line: anti-gangliosides mAbs (-). (**B**) mRNA expression of *St8sia1* in RAW264.7 cells and MC3T3 E1 cells on days 0, 7, 14, and 21 after induction to mature osteoblasts (*n* = 4). Data are expressed as mean ± SD. RAW264.7 cells without induction of osteoclastogenesis (Day 0) were used as a positive control in Figure 2A. Legend: ND, not detectable.

Expression levels of b-series gangliosides (GD3, GD2, GD1b, and GT1b) in osteoblasts were analyzed by flow cytometry (Figure 1). These gangliosides were not detected in MC3T3 E1 cells before (Day 0) and after (Day 7) induction to mature osteoblasts (Figure 2A). Consistent with these results of flow cytometry, the GD3 synthase gene (*St8sia1*) was at the limit of detection in MC3T3 E1 cells before (Day 0) and after induction (Day 7, 14, and 21) to mature osteoblasts (Figure 2B).

### 2.2. Expression of b-series Gangliosides and GD3 Synthase Gene in RAW264.7 Cells and Primary Cultured Pre-Osteoclasts Derived from Mouse Bone Marrow Cells in the Presence or Absence of Receptor Activator of Nuclear Factor Kappa-B Ligand (RANKL)

**Figure 3:**
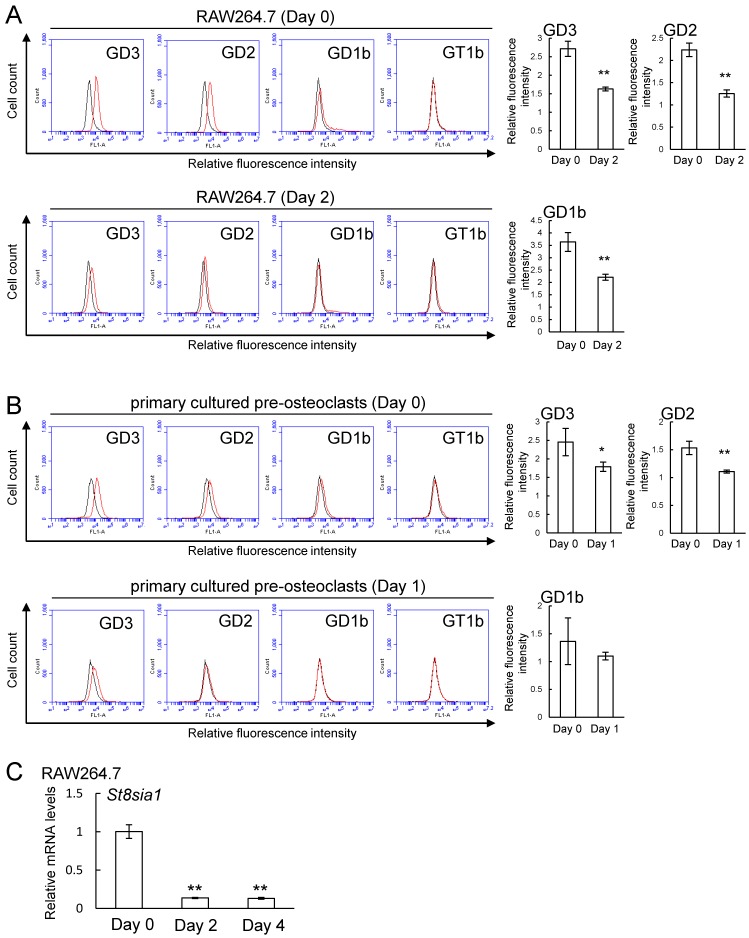
Expression of b-series gangliosides (GD3, GD2, GD1b, and GT1b) and GD3 synthase gene (*St8sia1*) in RAW264.7 cells and primary cultured pre-osteoclasts in the presence or absence of RANKL. (**A**) Expression of b-series gangliosides in RAW264.7 cells on days 0 and 2 after administration of RANKL (*n* = 3). (**B**) Expression of b-series gangliosides in primary cultured pre-osteoclasts on days 0 and 1 after administration of RANKL (*n* = 3). Red line: anti-gangliosides (GD3, GD2, GD1b, or GT1b) monoclonal antibodies (mAbs) (+), gray line: anti-gangliosides mAbs (-). The relative expression level (GD3, GD2, and GD1b) was defined as the ratio of the fluorescence intensity for the anti-gangliosides (GD3, GD2, or GD1b) monoclonal antibodies (mAbs) (+) to the fluorescence intensity for the anti-gangliosides mAbs (-). (**C**) mRNA expression of *St8sia1* in RAW264.7 cells on days 0, 2, and 4 after administration of RANKL (*n* = 3). Data are expressed as mean ± SD. The single (*) and double asterisks (**) indicate *p* < 0.05 and *p* < 0.01, respectively.

Both RAW264.7 cells and primary cultured pre-osteoclasts expressed GD3, GD2, and GD1b, and GT1b was below the limit of detection in both cells (Figure 3A,B). The expression of these gangliosides except for GD1b in primary cultured pre-osteoclasts was significantly decreased after induction of osteoclastogenesis by RANKL (Figure 3A,B). Consistent with these results was that expression levels of GD3 synthase gene (*St8sia1*) were downregulated by RANKL administration (Figure 3C).

### 2.3. Attenuation of Loss of Femoral Cancellous Bone Mass with Aging in GD3 Synthase-Knockout (GD3S KO) Mice

**Figure 4:**
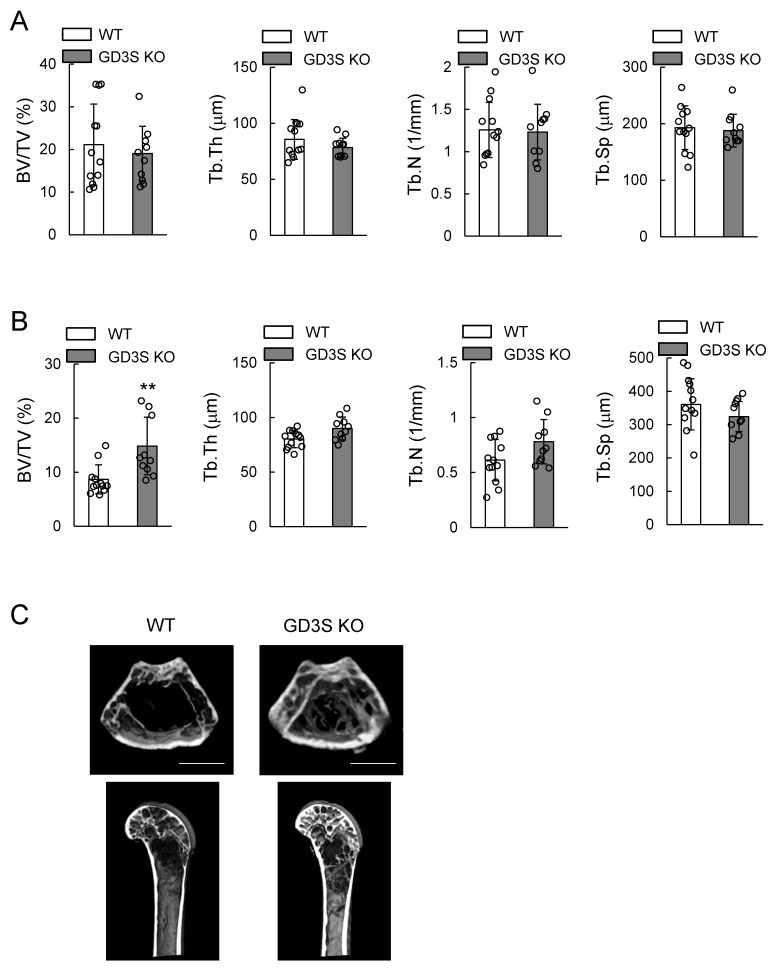
Attenuation of loss of femoral cancellous bone mass with aging in GD3S KO mice. Analysis of bone densitometry of the distal region of the femur in wild type (WT) and GD3S KO mice by three-dimensional micro-computed tomography (3D-μCT). (**A**) 15-week-old mice (male, *n* = 12 for WT, *n* = 10 for GD3S KO). (**B**) 40-week-old mice (male, *n* = 12 for WT, *n* = 10 for GD3S KO). Bone volume/total volume (BV/TV, %), trabecular thickness (Tb.Th, μm), trabecular number (Tb.N, 1/mm), and trabecular separation (Tb.Sp, μm) are presented. (**C**) Representative μCT images of femur in WT and GD3S KO mice at 40 weeks old. Results of WT mice (left) and GD3S KO mice (right) are shown. The scale bars are 1000 μm. Data are expressed as mean ± SD. The double asterisks (**) indicate *p* < 0.01.

No significant difference in the parameters of femoral cancellous bone mass was found at 15 weeks old between wild type (WT) and GD3S KO mice (Figure 4A). However, bone volume/total volume (BV/TV) in GD3S KO mice was significantly higher than that in WT mice at 40 weeks old (Figure 4B,C). Although trabecular thickness (Tb.Th) was not significantly different between 40-week-old WT mice and GD3S KO mice (*p* = 0.0503), Tb.Th in GD3S KO mice was higher than that in WT mice. Trabecular number (Tb.N) and trabecular separation (Tb.Sp) were not significantly different at 40 weeks old between WT and GD3S KO mice.

### 2.4. No Differences in Osteoblast Number in Femoral Cancellous Bone Between WT and GD3S KO Mice

**Figure 5:**
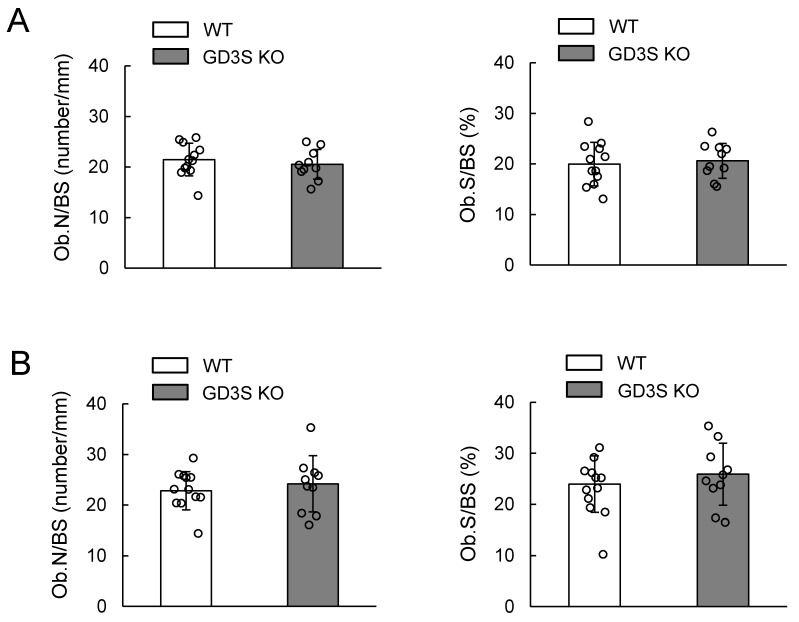
No effects of GD3 synthase deficiency on osteoblast numbers shown in vivo. (**A**) 15-week-old mice (male, *n* = 12 for WT, *n* = 10 for GD3S KO). (**B**) 40-week-old mice (male, *n* = 12 for WT, *n* = 10 for GD3S KO). Osteoblast numbers/bone surface (Ob.N/BS, number/mm) and osteoblast surface/bone surface (Ob.S/BS, %) are presented. Data are expressed as mean ± SD.

Bone formation parameters such as osteoblast numbers/bone surface (Ob.N/BS) and osteoblast surface/bone surface (Ob.S/BS) were almost equivalent between 15- and 40-week-old WT and GD3S KO mice (Figure 5), indicating that b-series gangliosides are not involved in bone formation.

### 2.5. Decrease of Bone Resorption Parameters by GD3 Synthase Deficiency

**Figure 6:**
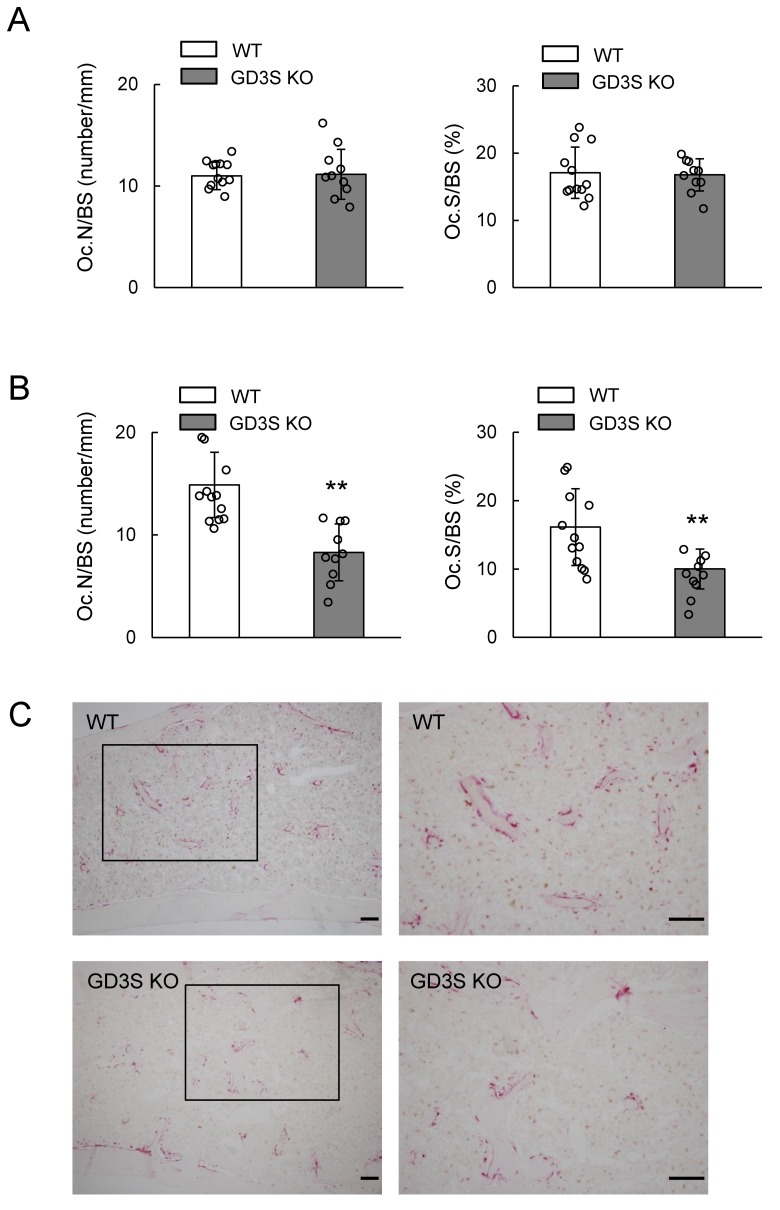
Decrease in bone resorption parameters by GD3 synthase deficiency in vivo. (**A**) 15-week-old mice (male, *n* = 12 for WT, *n* = 10 for GD3S KO). (**B**) 40-week-old mice (male, *n* = 12 for WT, *n* = 10 for GD3S KO). Osteoclast numbers/bone surface (Oc.N/BS, number/mm), and osteoclast surface/bone surface (Oc.S/BS, %) are presented. (**C**) Images of tartrate-resistant acid phosphatase (TRAP) staining of femoral cancellous bone. Results for WT mice (upper) and GD3S KO mice (lower) are shown. Right images are higher magnifications of the boxed areas in the images on the left. The scale bars are 100 μm. Data are expressed as mean ± SD. The double asterisks (**) indicate *p* < 0.01.

**Figure 7:**
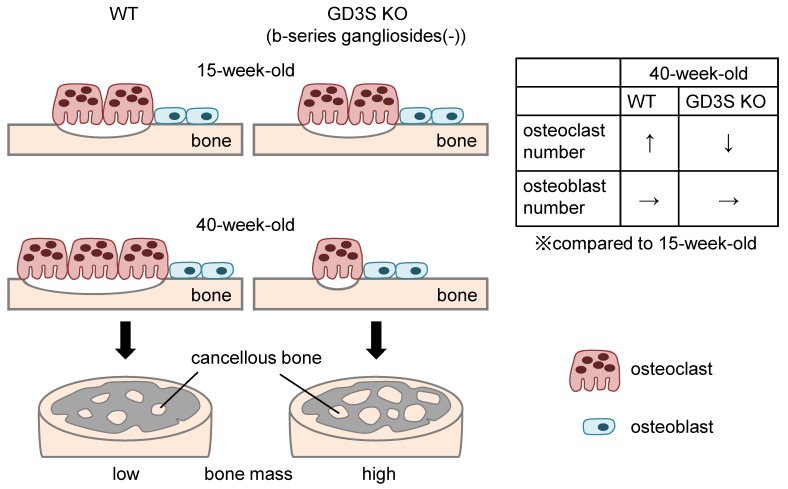
Schematic illustration of the proposed regulation of bone metabolism by b-series gangliosides.

No differences in osteoclast numbers/bone surface (Oc.N/BS) (WT, 11.0 ± 1.3; GD3S KO, 11.2 ± 2.5) and osteoclast surface/bone surface (Oc.S/BS) (WT, 17.1 ± 3.8; GD3S KO, 16.8 ± 2.4) were found between WT and GD3S KO mice at 15 weeks old (Figure 6A). However, Oc.N/BS (WT, 14.9 ± 3.5; GD3S KO, 8.3 ± 2.8) and Oc.S/BS (WT, 16.1 ± 5.5; GD3S KO, 10.0 ± 2.9) were significantly lower in GD3S KO mice than in WT mice at the age of 40 weeks (Figure 6B). All these results are summarized in a schema in Figure 7.

## 3. Discussion

In this study, we present the idea that deficiency of GD3 synthase in mice attenuates bone loss with aging (Figure 7). Although b-series gangliosides were not expressed in osteoblasts, these gangliosides were detected in pre-osteoclasts such as RAW264.7 and primary cultured pre-osteoclasts derived from mouse bone marrow cells. Furthermore, 3D-μCT and bone histomorphometric analyses using GD3S KO mice revealed that deficiency of GD3 synthase in mice suppressed bone resorption, leading to attenuation of a decrease in bone mass with aging.

3D-μCT analysis revealed that BV/TV in 40-week-old WT mice was reduced by 59% with aging (15 weeks old, 21.1 ± 9.5; 40 weeks old, 8.7 ± 2.7). On the other hand, BV/TV in 40-week-old GD3S KO mice was reduced by 23% with aging (15 weeks old, 19.1 ± 6.4; 40 weeks old, 14.8 ± 5.3). These results suggest that genetic deletion of GD3 synthase attenuated a decrease in bone mass with aging.

It has been reported that leptin secretion is disturbed by deficiency of b-series gangliosides, leading to a reduction in serum leptin levels in GD3S KO mice [7]. Leptin plays essential roles in the regulation of the mass of adipose tissue and body weight, and lack of leptin causes obesity [21,22,23]. Leptin is also known to increase bone resorption by the sympathetic nervous system [24]. In this study, we observed that body weight as well as bone mass in GD3S KO mice was higher than that in WT mice at the age of 40 weeks (supplementary Figure S1). Furthermore, bone resorption parameters in GD3S KO mice were lower than those in WT mice at the age of 40 weeks. These results suggest that lacking GD3 synthase reduces serum leptin levels, resulting in the attenuation of bone resorption. To determine whether secretion of leptin regulated by GD3 synthase is involved in bone metabolism, it is necessary to confirm serum levels of leptin in WT and GD3S KO mice, as we have reported previously [7], and examine whether injection of leptin into GD3S KO mice increases bone resorption with aging up to the same levels as WT mice.

Some studies have reported that gangliosides regulate differentiation of MSCs and tendon stem cells into osteoblasts [16,17,18,20]. For instance, it has been reported that GD1a plays important roles in the regulation of osteoblast differentiation of MSCs through the activation of epidermal growth factor receptor signaling [16,17]. GM1 also promotes osteoblastic differentiation of tendon stem cells by suppression of the phosphorylation of platelet-derived growth factor receptor-β (PDGFR-β) [20]. Furthermore, serum deprivation results in upregulation of the GM3 synthase gene as well as osteoblastic differentiation markers, such as Runx2 and osteocalcin [19]. Collectively, a-series gangliosides may be involved in the induction of osteoblastic differentiation. Levels of a-series gangliosides are known to be increased in GD3S KO mice [25] and we have confirmed that GD1a, among the a-series gangliosides, is expressed in MC3T3 E1 cells. However, elevation of a-series gangliosides in osteoblast cells may not be caused by a lack of GD3 synthase, since expression of b-series gangliosides was not observed in osteoblast cells and bone formation parameters were almost equivalent between WT and GD3S KO mice.

Although action mechanisms of gangliosides in bone metabolism were not fully understood in this study, our observations provide the first evidence of the role of GD3 synthase in bone metabolism in animals and suggest that altered ganglioside metabolism may underlie this phenomenon. In summary, we have demonstrated that a lack of GD3 synthase in mice results in the prevention of bone loss through the suppression of bone resorption based on a decrease in osteoclast number.

## 4. Materials and Methods

### 4.1. Mice

The generation of GD3S KO mice was carried out as described previously [11]. In brief, a targeting vector was used for gene knockout of GD3 synthase. The neo^r^ gene was inserted between the *Bal*I and *Acc*I sites in exon 1 of the GD3 synthase gene and a 9.5-kb gene fragment was used as a targeting vector. The diphtheria toxin A gene was attached to eliminate nonhomologous recombinants [11]. WT and GD3S KO mice were mated and the resultant heterozygotes were mated, and genotypes of the offspring were screened as described [11]. The mice were housed under a 12 h light/dark cycle, and water and food were provided ad libitum. All protocols for animal experiments were approved by the Aichi Gakuin University Animal Research Committee (approval number AGUD312; 29 October 2015) (Nagoya, Japan), and were carried out in accordance with the National Institutes of Health Guide for the Care and Use of Laboratory Animals (1966).

### 4.2. Cell Culture

The MC3T3 E1 mouse osteoblast-like cells and RAW264.7 mouse pre-osteoclast (monocyte/macrophage) cells were obtained from RIKEN BRC (Tsukuba, Japan) and American Type Culture Collection (Mnassas, VA, USA), respectively. The MC3T3 E1 cells, RAW264.7 cells, and mouse bone marrow cells isolated from long bones (femur and tibia) [26] were cultured in α-Minimum Essential Media with 10% fetal bovine serum and antibiotics (100 U/mL penicillin and 100 μg/mL streptomycin, Wako Pure Chemical Industries, Ltd., Osaka, Japan). Cells were maintained at 37 °C with 5% CO_2_ in a humidified incubator. They were then stained with trypan blue and the numbers of living and dead cells were counted using a hemacytometer.

### 4.3. Antibodies

Anti-GD3 monoclonal antibody (mAb) R24 was kindly provided by Dr. Lloyd J. Old at the Memorial Sloan-Kettering Cancer Center. Anti-GD2 mAb 220-51, anti-GD1b mAb 370, and anti-GT1b mAb 549 had been previously generated in Dr. Furukawa’s laboratory [27]. FITC-conjugated anti-mouse IgG and IgM were purchased from Affymetrix eBioscience (San Diego, VA, USA).

### 4.4. Induction to Mature Osteoblasts

The MC3T3 E1 cells were plated in 60 mm dishes. When cells were confluent, 50 μg/mL of ascorbic acid (Wako Pure Chemical Industries, Ltd., Osaka, Japan) and 5 mM β-glycerophosphate (Sigma-Aldrich, St. Louis, MO, USA) were added. The medium was changed every other day. After 7, 14, and 21 days of culture, cells were used for flow cytometry or quantitative real-time PCR.

### 4.5. In Vitro Osteoclast Induction

Mouse bone marrow cells were plated in 150 mm dishes and cultured with 10 ng/mL macrophage colony-stimulating factor (M-CSF, PeproTech, Inc., Rocky Hill, NJ, USA) for 3 days. The surface-attached cells were used as osteoclast precursors [26]. These cells were cultured with 10 ng/mL M-CSF and 50 ng/mL RANKL (PeproTech, Inc.). After 24 h of treatment with RANKL, the cells were used for flow cytometry.

### 4.6. Flow Cytometry

Cell surface expression of gangliosides was analyzed using an Accuri^TM^ C6 Flow Cytometer (BD Biosciences, San Jose, CA, USA) using various anti-ganglioside mAbs. The mAbs used in this study were anti-GD3 mAb (mouse IgG3, R24) (1:100), anti-GD2 mAb (mouse IgG1, 220-51) (1:100); anti-GD1b mAb (mouse IgM, 370) (1:100); anti-GT1b mAb (mouse IgM, 549) (1:100). The cells were incubated with individual antibodies for 1 h on ice and then stained with FITC-conjugated anti-mouse IgG (1:200) or IgM (1:200) for 45 min on ice. Control cells for flow cytometry were prepared using the secondary antibody alone. RAW264.7 cells without induction of osteoclastogenesis (Day 0) were used as a positive control in Figure 2A.

### 4.7. qPCR

Total RNA was extracted using an RNeasy Plus mini kit (Qiagen, Germantown, MD, USA). Reverse transcription was conducted with a high-capacity cDNA reverse transcription kit (Applied Biosystems, Carlsbad, CA, USA) and quantitative real-time PCR was performed using TaKaRa Thermal Cycler Dice Real Time System III with THUNDERBIRD SYBR qPCR mix kits (TOYOBO, Osaka, Japan). One μg total RNA was used for reverse transcription and the cycling conditions were 25 °C for 10 min, 37 °C for 120 min, and 85 °C for 5 min. The PCR cycling conditions were 95 °C for 10 min (pre-denaturation), 40 cycles at 95 °C for 15 sec (denaturation), and 60 °C for 1 min (extension). We evaluated the mRNA levels of *St8sia1* using the primers listed in Table 1. *Gapdh* was used for an internal control. The relative mRNA abundance for the selected genes with respect to the level of *Gapdh* mRNA was expressed as a ratio of *S*_MC Day 0, 7, 14, 21_ / *S*_RAW_ (Figure 2B) or *S*_RAW Day 2, 4_ / *S*_RAW Day 0_ (Figure 3C). *S*_MC Day 0, 7, 14, 21_ was the mRNA level for MC3T3 E1 cells before (Day 0) and after induction (days 7, 14, and 21) to mature osteoblasts. *S*_RAW_ was the mRNA level for RAW264.7 cells. *S*_RAW Day 0_ and *S*_RAW Day 2, 4_ were the mRNA level for RAW264.7 cells before (Day 0) and after administration of RANKL (days 2 and 4), respectively.

### 4.8. Three-Dimensional Micro-Computed Tomography (3D-μCT Analysis)

The distal region of the femur was scanned using 3D-μCT (CosmoScan R-mCT-GX-T1, RIGAKU, Tokyo, Japan) and the parameters of cancellous bone were analyzed by TRI/3D-BON (Ratoc, Tokyo, Japan) software. In brief, the scanning was initiated at 0.5 mm above the distal femoral growth plate, and a total of 75 consecutive 20-μm-thick sections were analyzed.

### 4.9. TRAP Staining

TRAP staining was conducted as described previously [28]. In brief, the slides (femur samples) were incubated in sodium acetate buffer (0.1 M, pH 5.0) containing naphthol AS-MX phosphate, Fast Red Violet LB Salt, and MnCl_2_ in the presence of sodium tartrate at 37 °C for 60 min for TRAP staining.

### 4.10. Bone Histomorphometric Analyses

Femur samples were fixed with 4% paraformaldehyde and decalcified in 10% ethylenediaminetetraacetic acid (EDTA) for three weeks, embedded in paraffin, and sectioned at 5 μm thickness. These slides were used for hematoxylin and eosin (HE) and TRAP staining. After staining with TRAP, osteoclast numbers on the bone surface and osteoclast surface on the bone surface were evaluated by scoring the TRAP-positive multi-nucleated cells on the bone surface as described previously [29]. Osteoblasts were stained with HE and osteoblast numbers on the bone surface and osteoblast surface on the bone surface were evaluated. The parameters were measured within an area of 0.8 mm^2^ (1.0 mm × 0.8 mm), with the closest and furthest edges being 2.0 and 3.0 mm distal to the growth plate of the proximal ends of the femur, respectively [29].

### 4.11. Statistical Analysis

All data were expressed as mean ± SD. Statistical significance was evaluated using a Student’s *t*-test (Figure 3) and a two-tailed Mann and Whitney U test (Figure 4, Figure 5 and Figure 6, and supplemental Figure S1) at *p* < 0.05; the single and double asterisks indicate *p* < 0.05 and *p* < 0.01, respectively.

## Figures and Tables

**Table 1:** Real-time PCR primers used in this study.

Target	Forward Primer	Backward Primer
*St8sia1*	5′-TGCTTTTTGCTAACCCCAAC-3′	5′-AAGGGCCAGAAGCCATAGAT-3′
*Gapdh*	5′-TGCACCACCAACTGCTTAG-3′	5′-GGATGCAGGGATGATGTTC-3′

## Supplementary Materials

Supplementary materials can be found at https://www.mdpi.com/1422-0067/20/11/2825/s1.
