# Supplementary material for: Deficiency of GD3 Synthase in Mice Resulting in the Attenuation of Bone Loss with Aging
Source: Int J Mol Sci. 2019 Jun 10;20(11):2825. doi: 10.3390/ijms20112825 (PMC6600367; doi:10.3390/ijms20112825)
Supplement: Supplementary file 1 [file ijms-20-02825-s001.zip › supplemental information (052819)/reGD3 KO mice project supplemental Figure 052819.pptx]

## Slide 1
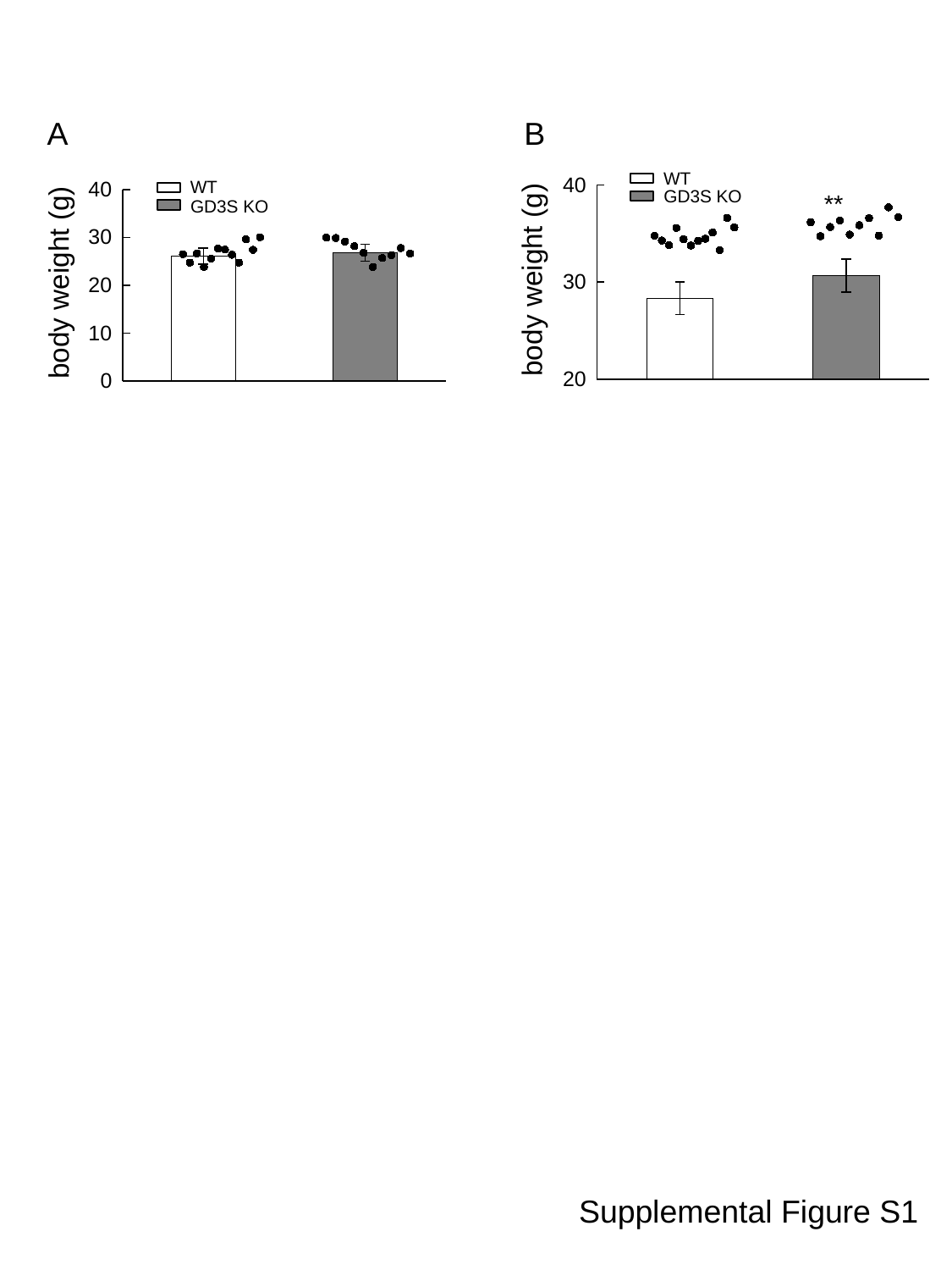

A
B
WT
### Chart
| Category | |
|---|---|
### Chart
| Category | |
|---|---|
### Chart
| Category | |
|---|---|
### Chart
| Category | |
|---|---|
### Chart
| Category | |
|---|---|
### Chart
| Category | |
|---|---|WT
**
GD3S KO
GD3S KO
body weight (g)
body weight (g)
Supplemental Figure S1
