# Supplementary material for: Deficiency of GD3 Synthase in Mice Resulting in the Attenuation of Bone Loss with Aging
Source: Int J Mol Sci. 2019 Jun 10;20(11):2825. doi: 10.3390/ijms20112825 (PMC6600367; doi:10.3390/ijms20112825)
Supplement: Supplementary file 1 [file ijms-20-02825-s001.zip › supplemental information (052819)/supplementalTEXT GD3 syn KO revision.docx]

**Supplemental information**

**Figure Legend**

**Supplemental Figure S1.** Body weight in WT and GD3S KO mice at the age of 15 or 40 weeks. (A) 15-week-old mice (male, n=12 for WT, n=10 for GD3S KO). (B) 40-week-old mice (male, n=12 for WT, n=10 for GD3S KO). Data are expressed as mean ± S.D. The double asterisks indicate *p* < 0.01.
